# A Simple Feedback Resistor Switch Keeps Latent HIV from Awakening

**DOI:** 10.1371/journal.pbio.0050025

**Published:** 2006-12-26

**Authors:** Richard Robinson

Upon entering a cell, a virus often becomes dormant, turning off its genes and laying low until awakened by some trigger from its environment. When that trigger is pulled, the virus quickly ramps up production of proteins through built-in positive-feedback loops that turn up gene transcription. (In positive feedback, production of something stimulates more production of that thing, resulting in exponential, or faster, growth.)

If the viral environment were perfectly regulated and viral gene expression perfectly silenced during latency, this system would be foolproof. But this is almost never the case—there is always noise and always the potential for some low level of erroneous transcription. This poses a problem for the virus—how does it prevent stray transcription from erupting into full-blown activation?

Certain bacterial viruses manage this problem by encoding intricate repressor circuits that efficiently block transcription. But animal viruses, specifically HIV, appear to lack similar repressor circuits. In a new study, Leor Weinberger and Thomas Shenk propose that some animal viruses, including HIV, regulate their potential for positive feedback and maintain latency by successively modifying and dissipating, or introducing a resistor into, the main activator of transcription.

HIV’s transcriptional activator, the *Tat* gene, is encoded in the HIV genome. Once *Tat* is transcribed, it can rapidly increase transcription not only of itself, but also of other genes that ultimately lead to viral replication. Thus, the Tat protein acts like a molecular switch, making it a likely target for regulating latency. In some kinds of molecular switches, the conversion between on and off states is regulated by self-oligomerization, or binding to several other identical molecules. The shape changes induced by binding or unbinding drive the complex into two different stable conformations. But, the authors found no experimental evidence for oligomerization of Tat; instead, both on and off forms appear to be monomers.

**Figure:**
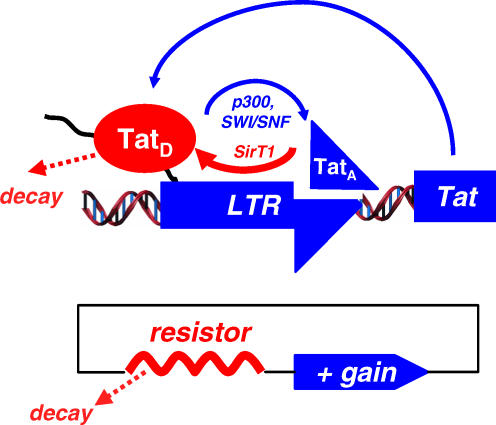
HIV Tat transactivates its own expression, but this requires conversion from deacetylated Tat (Tat_D_) to acetylated Tat (Tat_A_), a reaction that establishes a feedback resistor and allows the circuit to shut off

Other studies have shown that Tat is activated by the addition of an acetyl group—a functional group that is frequently added to (acetylation) or removed from (deacetylation) proteins to modify their properties—and that deacetylation inactivates Tat. Based on the known kinetics of both acetylation and deacetylation, the authors postulated that a resistor might exist in the Tat circuit. A simple mathematical model showed that the interconversion of the two forms, coupled with the known rate of breakdown of Tat, was sufficient to encode a resistor that explained Tat circuit shutoff and possibly the stability of HIV’s latent state.

In the Tat resistor model, as in the cell, Tat deacetylation occurs at a much faster rate than acetylation. Deacetylated (inactive) Tat can take one of two paths—reconversion into acetylated (active) Tat, or destruction of the protein by cellular machinery. When the appropriate conversion and destruction rates were fed into their model, activated Tat appeared briefly after a stray burst of transcription but quickly disappeared without breaking viral latency. This prediction of the model was then precisely replicated in cell culture experiments. An array of cell culture experiments perturbing the supposed Tat resistor was then performed. For example, inhibition of the deacetylating enzyme SirT1 induced Tat transcription activation in cells, further supporting the role of Tat acetylation in controlling viral dormancy. Finally, simulations under noisy conditions predicted that this simple resistor system was better able to resist environmental fluctuations than hypothetical oligomer-dependent switches, and cell-sorting experiments confirmed this prediction.

This simple switch, in which the deactivating reaction overpowers the activating reaction under most circumstances, acts as a “feedback resistor,” and its general features, the authors suggest, are likely to be found in other systems that must rapidly alternate between two states while resisting noise in the environment. Their model may also provide an explanation for some puzzling observations about Tat and HIV. Tat contains at least two acetylation sites that must both be deacetylated to turn off transcription. The authors propose this requirement may avoid making the off state so easy to reach that the virus remains dormant all the time. This model also helps explain why some HIV patients experience short “blips” of viral activity, despite relatively low viral concentration. According to the authors, these pulses of viral activation may be due either to random increases of Tat activity or to environmental inhibitors of the SirT1 enzyme, such as dihydrocoumarin, a natural flavoring agent found in clover.

